# Cystic mesenteric lymphangioma: A case report

**DOI:** 10.1016/j.ijscr.2019.07.051

**Published:** 2019-07-25

**Authors:** Ousmane Thiam, Papa Mamadou Faye, Abdou Niasse, Yacine Seye, Mohamadou Lamine Gueye, Ibrahima Sitor Sarr, Alpha Oumar Toure, Mamadou Seck, Mamadou Cisse, Madieng Dieng

**Affiliations:** a General Surgery Department at Dalal Jamm Hospital, Dakar, Senegal; b General Surgery Department at Aristide Le Dantec Hospital, Dakar, Senegal

**Keywords:** Cyst lymphagioma, Mesentery, Rare

## Abstract

**Introduction:**

Cystic lymphangioma is a benign malformative tumor. The abdominal localization is rare. The diagnosis is not easy in preoperative period. The surgery is the choice in the abdominal and symptomatic localization.

**Presentation of case:**

We report a 26 years old women. She consulted with left hypochondrial pain. The exam found left hyphochondrial swelling with 10 cm of diameter. The biologic screeming was normal. The ultrasound showed a multiloculated cyst which measured 130*80 mm. the CT scan showed a mesenteri cyst mass measured 15 cm. A fine needle aspiration cytology guided by abdominal ultrasound was realized and the cytology doesn’t show malignant cell. A median laparotomy found a mesenteric cystic mass measured 15 cm of diameter. A resection was realized. The histologic exam membranous fibrosis cyst limited by an endothelium.

**Conclusion:**

Mesenteric localization of cyst lymphagioma is rare. The surgery is safe and efficiency for the treatment.

## Highlights

• Abdominal Cyst lymphangioma.
• Mesenteric localization.
• Surgical treatment.
• Histological exam.

## 1 Introduction

This work has been reported in line with the scare criteria [1].

Cystic mesenteric lymphangioma is a benign peritoneal tumor. It is a spontaneous malformation composed by lymphatics vessels and lymphoid tissue [2,3]. It is the most common cystic mesenteric tumor [4]. It frequently appears in children, in 60% of cases before one year old [[5], [6], [7]] and is rare in adult people [8]. It localizes frequently in neck, facial, axillar and thoracic regions in 95%. The abdominal intra-peritoneal localization is out of ordinary [9]. The diagnosis is difficult in preoperative because of polymorphous clinical symptomatology. The imagery helps for the diagnosis [10]. However, histological exam is necessary for definitive diagnosis. Open surgery or laparoscopy is a good option for cystic lymphangioma, particularly in abdominal localization [9,11]. Though, percutaneous sclerosis is an efficient therapy, with less risks and could be the first option for this benign pathology [12].

## 2 Case presentation

**Fig. 1:**
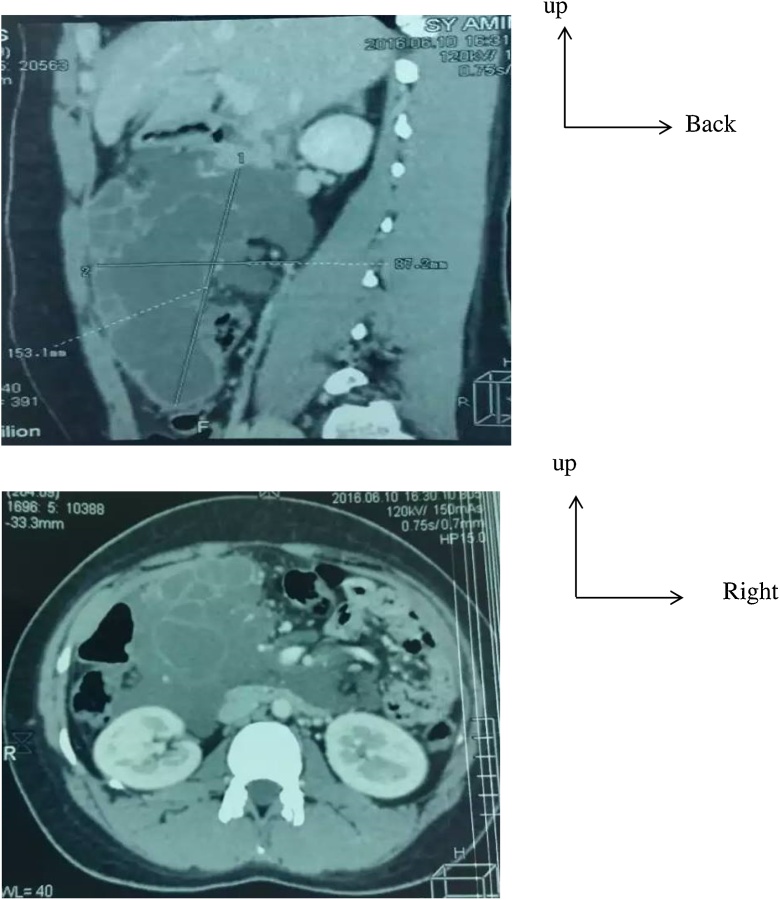
Abdominal CT scan with contrast showed cyst mass mesured 15 cm * 6,3 cm, subhepatic, with fluid density (arrow) encompassing multiples mesenteric vessels.

**Fig. 2:**
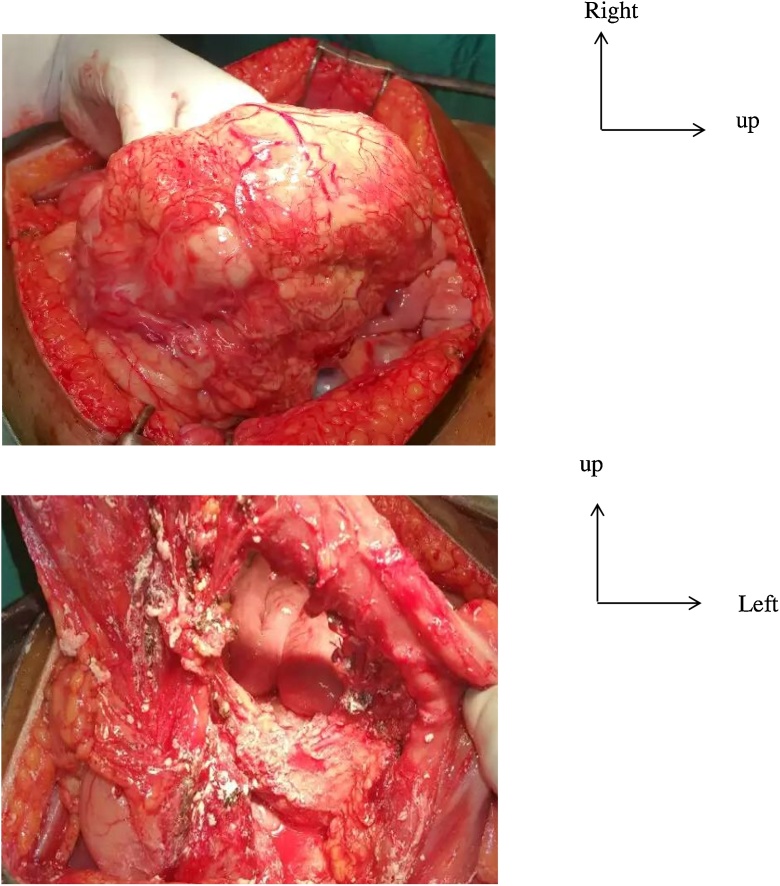
Preoperative view. A: aspect before resection. B: cavity of cyst mesenteric after resection.

The present case is a 26 years old woman, without medical antecedent. She consulted for abdominal pain in left hypochondrial and flank regions evolving for 72 h without transit disorders. The patient was afebrile with a normal hemodynamic monitoring. At the physical exam, there was a left hypochondrial swelling with 10 cm of diameter, slightly mobile. A biologic screening was performed with an elevated CRP at 96,6 mg, without elevation of leucocyte at blood count, a normal hepatic, pancreatic, and renal functions. The ACE and CA19-9 was normal respectively at 4 ng/ml and 32 UI/ml. The abdominal ultrasound showed a multiloculated cyst which measure 130*80 mm, with a thick wall localized at the infero-internal edge of the liver. The abdominal CT scan with contrast showed a mesenteric liquid mass, in the bilio-pancreatic intersession measured 15 cm and pushing back the adjacent organs, without secondary localization (Fig. 1, Fig. 2). A fine needle aspiration cytology guided by abdominal ultrasound was realized, and the cytologic result revealed multiple lymphocyte cells without malignant cells. The diagnosis of cystic mass compatible with a lymphangioma was retained.

A median laparotomy was performed, and the exploration found a mesenteric cystic mass measured 15 cm of diameter, resecable. A resection was realized, and the mass sent for a histological exam. The histological exam revealed a membranous fibrosis cyst measured 10*6 cm with multiple cavities limited by an endothelium with striated muscle tissue. This histological result confirmed the diagnosis of cystic lymphangioma.

## 3 Discussion

Cystic lymphangioma is a benign and rare tumor of lymphatics vessels. It is localized in subcutaneous tissue of face and neck (60%), limb (20%), thoracic (10%) and axillar regions. The abdominal (2–10%), and thoracic (5%) localizations are rare [13,14]. In abdomen, the lesions are preferentially localized in mesentery and the epiploon, and also found in the liver, spleen, pancreas, kidney, adrenal gland, large intestine and duedonum [[15], [16], [17], [18]]. In less than 10% of cases, lesions are diffuse with a peritoneal cystic lymphangiomatosis, similar to peritoneal carcinosis [25]. The physiopathology is a congenital default of connection between the primary lymphatics spaces and the central collector system [19]. The obstructed lymphatics vessels progressively dilate forming a multi-septated mass. The anatomic reports between this mass and adjacent structures are variable. The mesentery and retroperitoneal regions are preferentially attempt because of developed lymphatic system [10,20]. In mesenteric localization, incidence is 1/100,000 autopsies and 1–8/1,000,000 patients hospitalized. This frequency matches with 1/100,000 in adult patient and 1/20,000 in children [9,21,22]. The symptoms are poor, missing sometimes and non-specific, with a progressive evolution. During complications, as rupture, infection, hemorrhage, digestive volvulus the clinical presentation is an acute abdomen [3,4,10,23,24]. According to Losanoff and Kjossev, a classification based on the morphotype of lesions is necessary to optimize the surgical treatment [26] (Table 1). The diagnosis of an intra-abdominal cystic lesion requires a thorough investigation, to exclude a malignant process. The differential diagnosis is ovarian cystic, digestive duplicate, appendicular mucocele, pancreatic cystadenoma [24]. The medical imaging improved the diagnosis. The abdominal ultrasound is useful, non-irradiating, accessible and can make the diagnosis in antenatal period [20]. To ultrasound, a cystic liquid lesion, hypoechogenic, multilocular, with partition inside lesion, thick wall without vascularization in Doppler is found [24]. The abdominal CT scan is the gold standard for the diagnosis, giving more information for the configuration, size and the connection with the adjacent organ. The tumor is homogeneous, hypodense, thin partitions and not enhanced by the liquid contrast [24]. However, the partitions can be enhanced when there are thick [4]. The fatty content characteristic of chylous liquid can be objective by a negative density. In case of hemorrhage, we can have an elevation of density [24]. MRI is more specific for the content of the cyst, with low signal in T1 and high signal in T2 enhanced in effective T2 for liquid. For fatty content, a high signal in T1 and T2 decreasing in effective T2 [24]. A fine needle aspiration cytology, more invasive, precise the nature of the intra cystic liquid (chylous, serous, hemorrhagic) and the cytology exam confirms the present or not of lymphocyte cells and lipids. The bipedal lymphography was used for the retroperitoneal forms diagnosis but limited by the lymphatic communication rarely highlighted [27]. In situation of non-emergency, morphological assessment can be thorough. However, the surgery allows the treatment and the histological confirmation. The macroscopic examination reveals whitish or translucent cystic lesion. They can be unilocular (25%) or multilocular (75%). They are localized in mesentery, mesocolon, small intestine or in the root of meso. The resection of the cystic localization in the root of meso is difficult [24]. At the microscopic examination, three criterions are necessary for diagnosis:

- a cystic formation,
- the septa are constituted of a connective stroma with lymphoid tissue and striated muscle,
- the cyst is lined with a lymphatic endothelium with a positive D2-40 marker, specific to detection of lymphangiogenesis and lymphatic vessel invasion [20,[28], [29], [30]].

**Table 1:** Classification of mesenteric cystic lymphagioma accordind to Losanoff et Kjossev.

Types	Description et surgical possibilities
Type I	Pediculated with risk of torsion or volvulus. The resection is easy.
Type II	sessile, less mobile that may require a nearby organ sacrifice
Type III	Includes a retroperitoneal extension (damage to vital structures sometimes) rendering total exerese impossible
Type IV	Corresponds to extensive multi-organ damage

In case of accidental discovery of an asymptomatic lymphangioma cyst, therapeutic abstention with a regular follow up is recommended [20,28]. A spontaneous regression is observed in 1,6–16% of cases [20].

A total surgical excision is major to avoid recidivism and should be conservative for the adjacent organs because of the benign character of lymphangioma [20]. During the intervention, the lymphostasis must be good to minimize postoperative complications like lymphocele, chylous ascites [20]. The excision is realized either with a step by step ligature, or with the UltraCision or the LigaSure™ [20]. The recidivism rate is 40% after incomplete resection and 17% after a macroscopic complete resection [20,28].

The aspiration of the cyst content with or without injection of sclerosing product has variable long-term results: frequent recidivism, up to 100% for certain series [31]. This technique is used for symptomatic lesions, easily accessible and unresectable, without extensive intestinal lost [28].

Another therapeutic alternative is the per cutaneous sclerosis with sclerosing products like sulfate de bleomycin, doxycycline, OK 432, acetic acid, absolute alcohol [12]. The results are excellent, with 100% success with absolute alcohol in short series [32]. However, there are some limits for this technique: general anesthesia necessary even more for children, because of the pain induced by certain products; important inflammatory response may require a preventive intubation with monitoring in intensive care unit; post injection pain requiring morphine administration [12].

## 4 Conclusion

Cystic lymphangioma is a rare evolutive malformation benign tumor, requiring a complete surgical excision to minimize any recidivism. Aggressive surgery with extensive resection of adjacent organs to the cystic lymphangioma is prohibited because of its benign character. The positive diagnosis is histological. In case of surgical abstention, the patient should undergo a regular screening with ultrasound and a medical treatment. Persistent symptoms under medical treatment indicate a surgery. The per cutaneous treatment is reserved for cases requiring an extensive resection.

## Sources of funding

The authors declare they have received no funding for the preparation of this document

## Ethical approval

The ethical committee of the hospital gave the agreement to report this case.

## Consent

Written informed consent was obtained from the patient for publication of this case report and accompanying images

## Author’ contribution

Thiam Ousmane, Faye Papa Mamadou these authors participated in the making and correction of this document. all authors agreed with the publication of the document.

## Registration of research studies

Researchregistry4759.

## Guarantor

Papa Mamadou Faye.

## Provenance and peer review

Not commissioned, externally peer-reviewed.

## Declaration of Competing Interest

The authors declare no conflict of interest.

## References

[1] Agha R.A., Borrelli M.R., Farwana R., Koshy K., Fowler A., Orgill D.P., For the SCARE Group (2018). The SCARE 2018 statement: updating consensus Surgical CAse REport (SCARE) guidelines. Int. J. Surg..

[2] De Perrot M., Rostan O., Morel Le Coultre C. (1998). Abdominal lymphangioma in adults and children. Br. J. Surg..

[3] Silvestre De Sacy V., Keilani K., Duron J. (1992). Lymphangiome du mésentère, Une cause rare de syndrome douloureux abdominal aigu chez l’adulte. J. Chir. (Paris).

[4] Dufay C., Abdelli A., Le Pennec V. (2012). Diagnostic et traitement des tumeurs mésentériques. J. Chir. Visc..

[5] El Murr T., Youssef P., Khairallah S. (2006). Lymphangiome kystique médiastinal géant : un cas clinique et revue de la littérature. J. Méd. Lib..

[6] Hornick J.L., Christopher D.M., Fletcher C.D. (2005). Intraabdominal cystic lymphangiomas obscured by marked superimposed reactive changes: clinicopathological analysis of a series. Hum. Pathol..

[7] Brennan T.D., Miller A.S., Chen S.Y. (1997). Lymphangiomas of the oral cavity: a clinicopathologic, immunohistochemical, and electron-microscopic study. Oral Maxillofac. Surg..

[8] Solomou E.G., Patriarheas G.V., Mpadra F.A. (2003). Asymptomatic adult cystic lymphangioma of the spleen: case report and review of the literature. Magn. Reson. Imaging.

[9] Steyaert H., Guitard J., Moscovici J. (1996). Abdominal cystic lymphangioma in children: benign lesions that can have a proliferative course. J. Pediatr. Surg..

[10] Kably A., Moumen M., Raissouni N. (2003). Le lymphangiome kystique du mésentère et de l’épiploon. A propos de deux cas. Gynecol. Obstet. Fertil..

[11] Merrot T., Chaumoitre K., Simeoni-Alias J. (1999). Abdominal cystic lymphangiomas in children. Clinical, diagnostic and therapeutic aspects: a propos of 21 cases. Ann. Chir..

[12] Gorincour G., Paris M., Aschero A. (2006). Percutaneous treatment of cystic lymphangiomas. Ann. Chir. Plast. Esth..

[13] Hancock B.J., St-Vil D., Luks F.I. (1992). Complications of lymphangiomas in children. J. Ped. Surg..

[14] Icard P., Le Rochais J.P., Galateau F. (1998). Les lymphangiomes kystiques du médiastin. A propos de 3 cas, revue de la littérature. Ann. Chir..

[15] Hamdi A., Nouri A., Selmi M. (1993). Le lymphangiome kystique abdominal de l’enfant. Ann. Chir..

[16] Ros P.R., Olmsted W.W., Moser R.P. (1987). Mesenteric and omental cysts: histologic classification with imaging correlation. Radiology.

[17] Takiff H., Calabria R., Yin L. (1985). Mesenteric cysts and intra-abdominal cystic lymphangiomas. Arch. Surg..

[18] Gerosa Y., Bernard B., Lagneau M. (1993). Lymphangiome kystique du duodénum révélé par une hémorragie digestive et associé à une entéropathie exsudative. Gastroentérol. Clin. Biol..

[19] Zogo A., Tosi D., Protuese D. (1997). Cystic lymphangioma of the transverse mesocolon simulating neoplasm of the pancreatic tail. Ann. Ital. Chir..

[20] Bezzola T., Bülher L., Chardot C. (2008). Le traitement chirurgical du lymphangiome kystique abdominal chez l’adulte et chez l’enfant. J. Chir..

[21] Kurtz R.J., Heimann T.M., Beck A.R. (1986). Mesenteric and retroperitoneal cysts. Ann. Surg..

[22] Chung M.A., Brandt M.L., St-Vil D. (1991). Mesenteric cysts in children. J. Pediatr. Surg..

[23] Mordi A., Rabii K., Hameed E. (2012). Intestinal obstruction complicating a mesenteric cystic lymphangioma. J. Chir. Visc..

[24] Mabrut J.Y., Grandjean J.P., Henry L. (2002). Les lymphangiomes kystiques du mésentère et du méso-côlon. Prise en charge diagnostique et thérapeutique. Ann. Chir..

[25] Deshmukh S.S., Oak S.N., Karmarkar S.J. (1993). Mesenteric lymphangiomatosis in children: a distinct clinico-pathological entity. Pediatr. Surg. Int..

[26] Losanoff J.E., Kjossev K.T. (2005). Mesenteric cystic lymphangioma : unusual cause of intraabdominal catastrophe in an adult. Int. J. Clin. Pract..

[27] Vital J.L., Guivarc’h M., Mouchet A. (1975). A propos d’un cas de lymphangiome kystique rétropéritonéal opacifié par lymphographie. Ann. Radiol..

[28] Verdin V., Seydel B., detry O. (2010). Le lymphangiome kystique du mésentère. Cas clinique du mois. Rev. Med. Liège.

[29] Weeda V.B., Booij K.A.C., Aronson D.C. (2008). Mesenteric cystic lymphangioma: a congenital and an acquired anomaly? Two cases and a review of the literature. J. Pediatr. Surg..

[30] Okamoto D., Ishigami K., Yoshimitsu K. (2009). Hemorrhagic mesenteric cystic lymphangioma presenting with acute lower abdominal pain: the diagnostic clues on MR imaging. Emerg. Radiol..

[31] Alqahtani A., Nguyen L.T., Flageole H. (1999). 25 years’ experience with lymphangiomas in children. J. Pediatr. Surg..

[32] Puig S., Aref H., Brunelle F. (2003). Double-needle sclerotherapy of lymphangiomas and venous angiomas in children: a simple technique to prevent complications. AJR.

